# One year mortality of patients treated with an emergency department based early goal directed therapy protocol for severe sepsis and septic shock: a before and after study

**DOI:** 10.1186/cc8138

**Published:** 2009-10-21

**Authors:** Michael A Puskarich, Michael R Marchick, Jeffrey A Kline, Michael T Steuerwald, Alan E Jones

**Affiliations:** 1 Department of Emergency Medicine, Carolinas Medical Center, 1000 Blythe Blvd, Charlotte, North Carolina 28203, USA

## Abstract

**Introduction:**

Early structured resuscitation of severe sepsis has been suggested to improve short term mortality; however, no previous study has examined the long-term effect of this therapy. We sought to determine one year outcomes associated with implementation of early goal directed therapy (EGDT) in the emergency department (ED) care of sepsis.

**Methods:**

We performed a longitudinal analysis of a prospective before and after study conducted at a large urban ED. Adult patients were enrolled if they had suspected infection, 2 or more systemic inflammatory response criteria, and either systolic blood pressure (SBP) <90 mmHg after a fluid bolus or lactate >4 mM. Exclusion criteria were: age <18 years, no aggressive care desired, or need for immediate surgery. Clinical and outcomes data were prospectively collected on consecutive eligible patients for 1 year before and 2 years after implementing EGDT. Patients in the pre-implementation phase received non-protocolized care at attending physician discretion. The primary outcome was mortality at one year.

**Results:**

285 subjects, 79 in the pre- and 206 in the post-implementation phases, were enrolled. Compared to pre-implementation, post-implementation subjects had a significantly lower ED SBP (72 vs. 85 mm Hg, *P* < 0.001) and higher sequential organ failure assessment score (7 vs. 5, *P* = 0.0004). The primary outcome of 1 year mortality was observed in 39/79 (49%) pre-implementation subjects and 77/206 (37%) post-implementation subjects (difference 12%; *P* = 0.04).

**Conclusions:**

Implementation of EGDT for the treatment of ED patients with severe sepsis and septic shock was associated with significantly lower mortality at one year.

## Introduction

The rate of hospitalizations due to severe sepsis doubled during the past decade with estimates indicating that approximately 750,000 persons are affected annually in the USA [1]. Age-adjusted population-based mortality from severe sepsis appears to be increasing and sepsis currently ranks as the 10^th ^leading cause of death in the USA [2]. Although much of the therapy for severe sepsis occurs in intensive care units (ICU), as many as 500,000 cases of severe sepsis are initially managed in emergency departments (EDs) annually, with an average ED length of stay of five hours [3]. These data underscore the importance of ED diagnosis and therapeutic intervention for severe sepsis.

Published meta-analytic data suggest a significant survival benefit associated with the use of an early quantitative resuscitation strategy targeting explicit resuscitation endpoints in patients with sepsis [4]. The Surviving Sepsis Campaign international consensus guidelines for the management of severe sepsis and septic shock make a grade B recommendation for the routine use of early quantitative resuscitation [5]. The only prospective randomized trial of quantitative resuscitation in the ED was performed by Rivers and colleagues [6], which demonstrated that early goal-directed therapy (EGDT) resulted in a decrease in absolute in-hospital mortality of 16%. Since the report by Rivers and colleagues, numerous investigators have prospectively demonstrated that early identification and early quantitative resuscitation of severe sepsis using EGDT in the ED is both feasible and associated with improved hospital survival in clinical (non-research) settings [7-10].

We are aware of no previously published data that measures the long-term impact afforded by implementation of an early quantitative resuscitation strategy for severe sepsis. In the present study, we sought to test the hypothesis of a significant mortality reduction at one year among patients treated with EGDT in the ED compared with patients treated before protocol implementation.

## Materials and methods

### Study design and setting

We performed a longitudinal analysis of patients enrolled in a prospective before and after study of the clinical effectiveness of EGDT for the early treatment of severe sepsis and septic shock in the ED [9]. All patients were enrolled in the ED at Carolinas Medical Center, an urban 800-bed teaching hospital with more than 100,000 patient visits per year. The ED is staffed by emergency medicine resident physicians supervised by board-certified emergency medicine attending physicians. This study was approved and informed consent waived by the institutional review board and privacy board of Carolinas Healthcare System.

### Treatment protocol

Our EGDT protocol and the clinical impact of its implementation has been previously reported in detail [9]. In brief, our protocol was the similar to that of Rivers and colleagues [6] in that our early resuscitation targeted three physiologic endpoints: central venous pressure (CVP), mean arterial pressure (MAP) and central venous oxygen saturation (ScvO_2_) using various stepwise therapeutic interventions to achieve predefined values of each endpoint. Our protocol differed from that described by Rivers and colleagues in that: it was executed only by ED physicians and nurses that were providing clinical care to the patient; and it was initiated in the ED and care was subsequently transitioned to the ICU during the resuscitation period. The use of serum lactate concentrations to screen for global hypoperfusion was encouraged but not mandated by the protocol. Because this quantitative resuscitation protocol was implemented relatively early after the original study (in 2005), no faculty or trainees at our hospital had prior experience with the use of a structured quantitative resuscitation protocol for sepsis.

### Study subjects

Eligible subjects were identified by board-certified emergency physicians in the ED, and inclusion criteria were identical for both phases: age 18 years and older; suspected or confirmed infection; two or more systemic inflammatory response syndrome (SIRS) criteria [11] (heart rate >90 beats per minute, respiratory rate >20 breaths per minute, temperature >38 or <36°C, white blood cell count >12,000 or <4000 cells/mm^3 ^or >10% bands); systolic blood pressure below 90 mmHg or MAP below 65 mmHg after a 20 ml/kg isotonic fluid bolus OR anticipated need for ICU care and a serum lactate concentration of 4.0 mM or higher. Exclusion criteria were: age less than 18 years; need for immediate surgery with an anticipated departure to the operating room in less than six hours; absolute contraindication for a chest central venous catheter. As our intent was to measure the potential impact of the early resuscitation program, subjects who did not survive the first six hours of early resuscitation (e.g. care was withdrawn early or the subject dies prior to the initial six hours of resuscitation) were excluded *post-hoc *from both groups.

The pre-implementation phase encompassed 13 months, from August 2004 to September 2005. During this time emergency physicians identified candidates with the inclusion and exclusion criteria and entered patient data in real-time on a computer in the ED using a secure web-based electronic collection form [12]. In this phase, care was provided by emergency physicians at their discretion and no formal protocol was utilized. The post-implementation phase encompassed two years, from November 2005 to October 2007. During this phase identification of an eligible patient triggered an alphanumeric page to ancillary staff (ED and ICU charge nurses, respiratory therapist, pharmacy, bed management) and both the protocol quality assurance nurse and physician. In all cases, the ED physicians and staff identified the patients, initiated the resuscitation protocol, placed the central venous catheter, and followed the protocol until a bed in the ICU was available for patient transfer. At the time of patient transfer from the ED to ICU, clinical care was transferred from the ED physicians to the admitting physicians.

### Data analysis and outcomes

The primary outcome was one-year mortality rate. The admission date of the index visit for sepsis was used as the baseline date and our query was intended to confirm deaths within one year after the baseline date. We assessed for the primary outcome through a two-tiered method. The first tier was to search our healthcare system's electronic medical record database, which contains all patient encounters within a healthcare system of 23 acute care hospitals and 57 outpatient care facilities in North and South Carolina, USA, using methods we have previously described [13]. Using this process the primary outcome was confirmed if: the subject had a documented visit to a healthcare facility more than one year after the baseline date; or the subject had a death confirmed via both an 'expired' discharge status and a physician documented death note in a healthcare facility within one year of the baseline date. For subjects without a primary outcome using the electronic medical records, we then progressed to a social security death index (SSDI) search. We searched the master SSDI using every combination of first, middle and last name, and social security number [14]. Both of the above searches (medical record and SSDI) were completed at 15 months or more after enrollment. If this two-tiered method did not establish a valid outcome of alive or dead, we assumed the subject to be alive.

Additional data collected included demographics and clinical variables, hospital resources utilized including the number of both ICU and hospital days. For both hospital and ICU days, if a patient spent any amount of time during the 24-hour period of one day in the ICU or hospital, it was counted as a full day. We also followed any sepsis-specific therapies that were administered, such as parenteral corticosteroids and activated protein C. The sequential organ failure assessment (SOFA) score was calculated in all patients at the time of identification [15].

Continuous data are presented as means ± standard deviation, and when appropriate were compared for statistical differences using unpaired t-tests or Mann Whitney U tests. Categorical data are reported as proportions rounded to the nearest whole number and associated 95% confidence intervals (CI) and where applicable tested for significance using Chi squared or Fisher's exact tests. The Kaplan-Meier survival estimates and log-rank test for comparison were used for time-to-death analysis. Cox proportional hazards regression was performed in order to determine hazard ratios for death at one year. The overall intent of the hazards regression was to determine the hazard ratios for death of patients who were treated with EGDT while controlling for other important variables that were found to have significant differences between our groups in the bivariate analysis. For all statistical tests *P *< 0.05 were considered significant.

## Results

We enrolled 293 patients in the current study. Six subjects in the post-implementation phase and two patients in the pre-implementation phase were excluded *post hoc *for not receiving the full six hours of early resuscitation (all died in <6 hours). Thus, we analyzed 79 subjects in the pre-implementation phase and 206 in the post-implementation phase. Table 1 shows the demographics, co-morbidities, clinical variables, severity of illness score, and source of suspected infections between the groups. The groups were well matched for demographics and co-morbidities. Subjects in the post-implementation phase had variables suggesting a higher severity of illness with a lower initial systolic blood pressure, higher initial respiratory rate and higher initial SOFA score, as compared with pre-implementation subjects.

**Table 1:** Patient demographics, clinical characteristics, and physiological measurements

	*Pre-implementation*	*Post-implementation*	
*Variable*	n = 79	n = 206	P *value*
**Age (mean years ± SD)**	58 ± 16	56 ± 18	0.58
**Race n, (%)**			
Caucasian	40 (51)	110 (54)	0.68
Black American	38 (48)	84 (41)	0.27
**Gender n, (%)**			
Male	47 (59)	101 (49)	0.12
Female	32 (41)	105 (51)	0.12
**Co-morbidities n, (%)**			
Diabetes mellitus	23 (29)	53 (26)	0.56
COPD	12 (15)	41 (20)	0.37
HIV	8 (10)	24 (12)	0.74
DD-End-stage renal disease	25 (32)	28 (14)	0.0008
Cancer	9 (11)	33 (16)	0.33
Organ transplant	3 (4)	4 (2)	0.4
Indwelling vascular line	7 (9)	27 (13)	0.33
Nursing home resident	18 (23)	39 (19)	0.47
Do not resuscitate	5 (6)	5 (2)	0.14
**ED vital signs (mean ± SD)**			
Lowest SBP (median, (IQR) mmHg)	85 (73-91)	72 (65-79)	< 0.0001
Highest pulse (beats/min)	118 ± 27	120 ± 25	0.5
Highest RR (breaths/min)	26 ± 9	30 ± 11	0.008
Highest temperature (°F)	101 ± 3	100 ± 3	0.04
Lowest O2 saturation (%)	94 ± 7	92 ± 7	0.35
Lowest CVP (mmHg)	-	7 ± 4	-
Highest CVP (mmHg)	-	13 ± 6	-
Lowest ScVO2 (%)	-	67 ± 13	-
Highest ScVO2 (%)	-	80 ± 11	-
**ED SOFA score (mean ± SD)**	5 ± 3	7 ± 4	0.0004
Lactate level** (mM, mean ± SD)	5 ± 3	4 ± 3	0.03
Suspected source of infection* n, (%)			
Pulmonary	25 (32)	89 (43)	0.08
Urinary tract	21 (27)	58 (28)	0.8
Intra-abdominal	14 (1)	41 (20)	0.52
Skin/soft tissue	13 (20)	27 (13)	0.22
Blood (bacteremia)	2 (3)	21 (10)	0.03
Unknown	12 (15)	16 (8)	0.07

*Some patients had more than one suspected source, thus the total is more than 100%.

**Lactate was only available in 33 of 79 before group patients and 193 of 206 after group.

COPD = chronic obstructive pulmonary disease; CVP = central venous pressure; DD = dialysis dependent; ED = emergency department; HIV = human immunodeficiency virus; IQR = interquartile range; O2 = oxygen; RR = respiratory rate; SBP = systolic blood pressure; ScvO2 = central venous oxygen saturation; SD = standard deviation; SOFA = sequential organ failure assessment.

Table 2 shows the resuscitative interventions utilized in the initial six hours of EGDT between the groups. Patients in the post-implementation group were intubated more frequently, received a significantly larger crystalloid volume and more frequent infusion of vasopressors, as compared with the pre-implementation group. We observed no significant differences in the rate of packed red blood cell transfusion, dobutamine administration, or median time to antibiotic administration. We also observed an increase in both the mean ICU and hospital length of stay in the post-implementation group.

**Table 2:** Resuscitation interventions utilized in the initial six hours

*Intervention*	*Before group* n = 79	*After group* n = 206	P value*
**Endotracheal intubation n, (%)**	7 (9)	55 (27)	0.0006
**Crystalloid volume (median, (IQR) liters)**	2.0 (1.0-3.4)	5.0 (3.8-7.2)	< 0.0001
**Vasopressor administration n, (%)**	27 (34)	149 (72)	< 0.0001
**Dobutamine administration n, (%)**	1 (1)	9 (4)	0.22
**PRBC transfusion n, (%)**	1 (1)	13 (6)	0.07
**Other**			
Time to initial antibiotics (median, (IQR) minutes)	85 (50-190)	90 (55-156)	0.62
Steroid administration n, (%)	5 (6)	88 (43)	< 0.0001
Activated protein C n, (%)	3 (4)	5 (2)	0.54
**ICU length of stay, days**	2 ± 3	4 ± 5	< .0001
**Total hospital days, days**	8 ± 6	10 ± 9	.0670

ICU = intensive care unit; IQR = interquartile range; PRBC = packed red blood cell.

**Figure 1:**
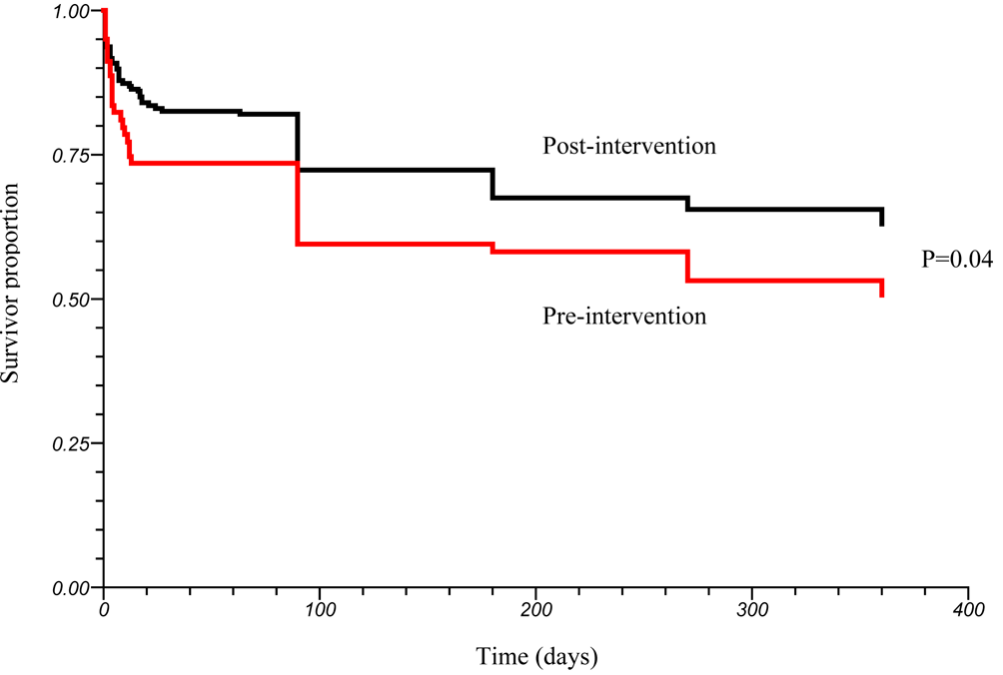
Kaplan Meier survival curve comparing survival of patients in the pre-implementation and post-implementation phases. The *P *value shown was derived from the log-rank test.

**Figure 2:**
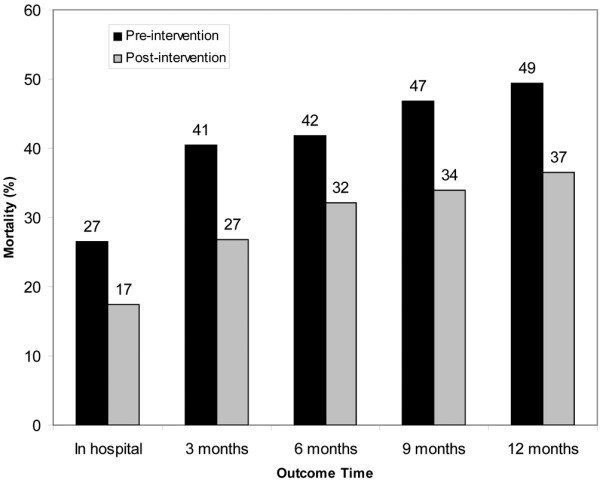
Mortality rates over the course of the first year after the index emergency department visit for severe sepsis or septic shock.

The primary outcome of one-year mortality was observed in 39 of 79 (49%) patients in the pre-implementation phase and 77 of 206 (37%) patients in the post-implementation phase. Valid outcome was unable to be reliably established in two patients in the pre-implementation and four patients in the post-implementation phases. All of these patients were coded as 'alive' for the analysis. The Kaplan-Meier survival estimate (Figure 1) showed significant differences between the groups for the primary outcome of one-year morality (log rank test *P *= 0.04). There was an increase in mortality during the year after treatment with EGDT in both the pre-implementation and post-implementation groups (Figure 2). The largest mortality increase was seen at the time point of three months after hospitalization in both groups. At one year after treatment, between 40% (post-implementation phase) and 50% (pre-implementation phase) of the subjects had expired.

Table 3 shows the results of the Cox proportional hazards regression analysis. Subjects who received EGDT were found to have a statistically significant reduction in risk of death at one year (Hazard ratio 0.55, 95% CI 0.35 to 0.87). Initial SOFA score was a predictor of one year mortality; however, other factors such as dialysis dependent end-stage renal disease and corticosteroid treatment were not predictors of one-year mortality.

**Table 3:** Results of Cox proportional hazards regression analysis

*Variable†*	Hazard ratio	95% CI	*P *value
Received EGDT*	0.55	0.35-0.87	0.01
Lowest ED SBP	1.0	0.99-1.01	0.59
Highest ED RR	1.0	0.99-1.01	0.55
DD-ESRD**	1.5	0.95-2.3	0.08
Initial SOFA score	1.1	1.05-1.2	0.004
Received steroids***	1.1	0.7-1.7	0.70

CI = confidence interval; DD ESRD = dialysis dependent end stage renal disease; ED = emergency department; EGDT - early goal directed therapy; RR = respiratory rate; SBP = systolic blood pressure; SOFA = sequential organ failure assessment.

* Patients in the pre-implementation group did not receive EGDT and those in the post-implementation group did receive EGDT.

** Refers to patient reported diagnosis established previous to index hospitalization.

*** Refers to patients who received systemic corticosteroids during the index hospitalization.

^† ^Dependent variable: one-year mortality.

Model Analysis

Log likelihood with all covariates = -613.

Deviance chi-squared = 26.9, degrees of freedom = 6, *P *= 0.0001.

## Discussion

In this study we document the one year outcomes of subjects treated with an EGDT algorithm for the management of severe sepsis and septic shock in the ED. At one year, we found a statistically significant 12% mortality reduction among subjects treated with the protocol suggesting a number needed to treat (1/absolute mortality reduction) of approximately eight persons to save one life for a year. This mortality reduction remained significant in a multivariable model that controlled for other potential explanatory variables. Furthermore, this mortality benefit was found among a group of patients with apparently higher severity of illness based on lower systolic blood pressures and higher sequential organ failure scores measured at enrollment.

We believe this report adds novel data to the early sepsis resuscitation literature. In the original EGDT study published by Rivers and colleagues, 60-day mortality was reported to be 57% in the standard therapy arm and 44% in the EGDT arm [6]. In a prospective observational study, Karlsson and colleagues reported the in-hospital and one-year mortality of severe sepsis in Finland [16]. Their findings are similar to our pre-implementation group with the same in-hospital mortality (28%) and a slightly lower one-year mortality of 41%. In the report by Karlsson and colleagues, all subjects with SIRS criteria and at least one with organ dysfunction were included. Our study required SIRS criteria and evidence of hypoperfusion (elevated lactate and/or hypotension after fluid challenge), which may account for the slight differences in outcomes noted. Also, the study by Karlsson and colleagues was observational and did not test implementation of a new treatment paradigm as did the present study. The authors do not mention EGDT and the incidence of its use in their study is not reported. Thus we believe the present report to be the first to document the long-term impact of an ED-based EGDT protocol on survival.

Our data indicate that subjects who are treated for severe sepsis and septic shock have a stepwise increase in mortality over the first year. This mortality increase over the first year was found in both phases of our study. It might be questioned why an early resuscitation would be associated with long-term mortality. One interpretation of this finding, as indicated in Figure 2, is that among those subjects in the post-implementation phase who derived the most benefit from the intervention were individuals who were the most 'salvageable' (i.e., those individuals who subsequently went on to survive to more than one year). Another possibility for our findings could be related to a Hawthorne effect, caused by heightened awareness of the clinical staff that resulted in a different response to post-implementation subject's clinical needs.

Our data also allow an inference into the expected one-year mortality among patients undergoing aggressive therapeutic intervention for sepsis using consensus recommendations [5], which is important for the purpose of designing future clinical trials incorporating longer range outcome assessment. Specifically, 40% of aggressively treated subjects are dead at one year after the index visit, suggesting a potential opportunity for targeted improvement, particularly for investigators designing trials that target longer term outcomes.

We found some important differences between the subjects in the pre- and post-implementation groups. There were significantly more subjects with dialysis dependent end-stage renal disease in the pre-intervention group (32% vs. 14%). Patients with end-stage renal disease who develop sepsis have been shown to have a higher mortality compared with the general population [17]. Also, significantly more subjects in the post-intervention group were treated with corticosteroids, a therapy which meta-analytic data have been suggested to have a beneficial effect on short-term mortality [18]. Both of these group differences could have an impact on the mortality benefit we observed. To address this concern we performed proportional hazards regression analyses, which revealed neither of these variables to be independent predictors of one-year mortality in our subjects.

The EGDT sepsis protocol comprises a resource intensive therapeutic intervention. Our data show a two-day increase in both ICU (statistically significant) and hospital length of stay (not-statistically significant). Our findings are in contrast to those of Rivers and colleagues who reported a non-significant 0.2 day difference in hospital length of stay between the control and EGDT group and did not report mean ICU length of stay. This increase in resources utilized in the ICU is a finding that deserves more investigation.

This report has several limitations that warrant discussion. First, this is a single-center study that was not conducted as a tightly controlled experimental investigation. As such, our results may not be generalizable to other populations. Second, therapies administered in the ED other than EGDT (e.g. antibiotics or steroids) or therapies administered after the EGDT period (e.g. during the first 72 hours of ICU care) may have contributed to the treatment effect we observed. Third, we used a two-tiered approach to establish one-year mortality in lieu of direct patient contact. Although we have previously published the validity of these methods [13,19] it is possible that our results might be different if a different follow-up method were used. Fourth, because our cohorts are not contemporaneous but actually divided along a time continuum, it is important to note that some of the study impact may be due to changes in technology, skill or other factors during the study period. Fifth, we did not measure physiological or severity of illness variables before and after the resuscitation in the post-implementation group. Thus it remains possible that some of the benefit demonstrated by the resuscitation was due to heightened awareness of the patient's illness. Finally, we did not quantify, explore, or exclude protocol deviations, because this study was designed to determine the impact of EGDT when implemented into a real-world clinical setting.

## Conclusions

Implementation of EGDT in the ED for the early treatment of severe sepsis and septic shock was associated with a significantly lower mortality at one year. This is the first large prospective study to suggest a long-term survival benefit associated with early and aggressive resuscitative care for sepsis.

## Key messages

• Early resuscitation of severe sepsis in the ED in a non-research setting was associated with a lower mortality at one year.

• The long-term survival association found with EGDT remained significant after adjusting for confounding in a multivariable model.

• Our results suggest a number needed to treat of eight subjects with EGDT to save one life at one year.

## Abbreviations

CI: confidence interval; CVP: central venous pressure; ED: emergency department; EGDT: early goal-directed therapy; ICU: intensive care unit; MAP: mean arterial pressure; ScvO2: central venous oxygen saturation; SIRS: systemic inflammatory response syndrome; SOFA: sequential organ failure assessment; SSDI: social security death index.

## Competing interests

Dr Jones has research support from Critical Biologics and Hutchinson Technology. Dr Kline is inventor on US patent 7,083,754. The remaining authors have no competing interests.

## Authors' contributions

AEJ conceived the study. AEJ MAP, MRM, MTS, and JAK designed the study. AEJ, MAP, JAK, MRM, and MTS collected the data and performed the statistical analysis. AEJ drafted the manuscript and all authors contributed significantly in revisions of the manuscript. All authors have read and approved the final manuscript.
